# Consequences of hazardous dietary calcium deficiency for fattening bulls

**DOI:** 10.1186/1751-0147-48-25

**Published:** 2006-12-08

**Authors:** Teppo Heinola, Elias Jukola, Päivi Näkki, Antti Sukura

**Affiliations:** 1 Department of Basic Veterinary Sciences, Faculty of Veterinary Medicine, University of Helsinki, P.O. Box 66, FI-00014 University of Helsinki, Finland; 2 LSO Foods Oy, P.O. Box 49, 01511 Vantaa, Finland

## Abstract

**Background:**

Deficient mineral supplementation on a feedlot farm resulted in severe clinical manifestations in fattening bulls. Animals mistakenly received only 60–70% of the recommended calcium intake, while simultaneously receiving twice the amount of phosphorus recommended. Thus, the dietary Ca/P ratio was severely distorted. After approximately six months on such a diet, four fattening bulls were euthanized because of severe lameness and 15% of other animals on the farm were having clinical leg problems. Veterinary consultation revealed the mistake in mineral supplementation.

**Methods:**

Fattening bulls were divided into three groups depending on the time of their arrival to the farm. This enabled the effect of mineral imbalance at different growth phases to be examined. After slaughtering, the bones of both front and hind limbs were macroscopically evaluated.

**Results:**

Over 80% of the animals with a calcium-deficient diet had at least one severe osteoarthritic lesion. The economic impact of the calcium deficiency was statistically significant.

**Conclusion:**

Calcium deficiency with distorted Ca/P ratio yielded a severe outbreak of osteoarthritis in fattening bulls. Calcium deficiency caused a more serious lesions in age group 5–12 months than age group 12–18 months. Besides causing obvious economic losses osteoarthritis is also a welfare issue for feedlot animals.

## Background

Lameness of fattening dairy and meat bulls is an animal welfare issue that also has significant economic consequences. Affected animals often suffer from osteoarthritis (OA) [1]. OA is a degenerative joint disease affecting the articular-epiphyseal cartilage complex. The aetiopathogenesis in growing bulls is variable, including trauma and osteochondrosis (OC) [1]. OC is a failure of endochondral ossification [2,3].

OC is believed to be multifactorial, but the exact risk factors are still under debate. Typical predisposing causes connected to the development of OC in cattle and swine are nutritional, environmental [4,5] and hereditary [6]. A rapid growth rate [7], which is linked to high-intensity feeding [8-10], is strongly associated with OC. Moreover, calcium or phosphorus deficiency or an imbalance of these minerals is reported to be related to OC [11]. Bulls growing in a hard-surface environment or on slatted floors tend to have more severe growth cartilage changes [4,10]. A higher risk is also associated with tie stall systems and lack of movement [12,13]. In addition, different kinds of traumas can lead to OC [5]. According to some studies, hereditary factors and gender of the animal may have an impact on development of OC [14,7,10]. Numerous interactions between different predisposing factors also exist.

Animals suffering from OA often show such clinical symptoms as lameness and unwillingness to move, fluid in affected joints and difficulty in standing up. Their gait is stiff and the lameness is frequently bilateral. Ruptures of the Achilles tendon have also been reported [8]. Medical and surgical therapies can be used on bulls with OA and OC, but the prognosis is poor [10].

Clinically, OC is most often seen in animals aged 14–22 months [8], and OA in older dairy bulls which may also have OC lesions [9]. OC as a failure of endochondral ossification is naturally associated with maturation and growth of the skeleton [4,10]. One study suggests that osteochondrotic changes start to emerge before weaning [15]. In any case, the interactions between age and exposures to predisposing factors are not fully understood.

In this case report, we describe the consequences of an accidental mineral deficiency on a feedlot farm. We analyse differences between exposure groups and estimate the economic losses due to calcium deficiency and OA.

## Materials and methods

### Case history

A Finnish dairy bull owner contacted the veterinarian because the animals were having an increasing number of leg problems. Affected animals were lame; they had difficulty in getting up and spent most of their time recumbent. The first symptoms were noticed about one month before contacting the veterinarian.

Four animals aged approximately 12 months were euthanized because they were unable to stand. Carcasses were sent to the slaughterhouse, where they were inspected by a veterinarian. The first animal had a rupture of the Achilles tendon with suppurative inflammation and an acute, bilateral aseptic inflammation of the stifle joints. The second animal had a bilateral Achilles tendon rupture. The third bull had a fractured front leg and aseptic inflammation of the stifle and elbow joints. The fourth animal had aseptic inflammation bilaterally of the elbow joint and the stifle joint and a ruptured Achilles tendon. The veterinarian sent a hind leg of the fourth animal the Finnish Food Safety Authority, Kuopio Research Unit, for pathological evaluation. Lesions in the hock joint were reported macroscopically to be typical for osteochondrosis.

At the time of the author's farm visit (TH); six animals had already been slaughtered due to severe lameness. At the visit, 16 of the 106 animals were found to have some kind of leg problems. Affected animals were lame and had different degrees of swelling of the joints, mainly in the hock and stifle joints.

**Figure 1:**
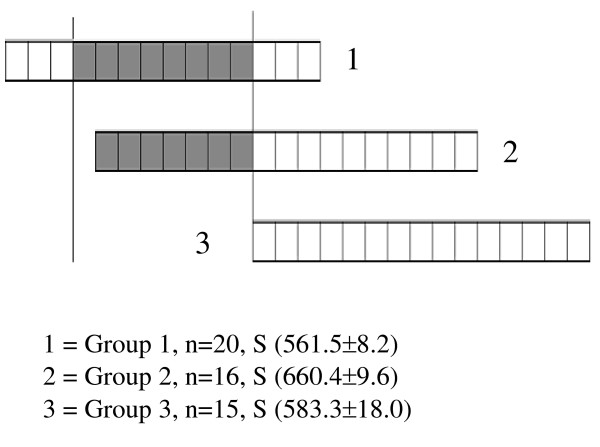
Time and duration of exposure to a low-calcium diet in different fattening groups. S( ) = mean age ± SE (days) of the animals in the group at the time of slaughter. Each box is equal to 30 days. The calcium-deficient period is indicated with shading.

The unit where leg problems emerged was for fattening of dairy bulls from the age of 6 months to slaughter. Target weight at 18 months was 330 kg. Minerals were added to the animals' drinking water. In this kind of system, calcium should also be provided in the ration. However, in this case, calcium was mistakenly not added, and thus, animals aged 6–18 months were calcium-deficient. At the time of the farm visit, confusion with the feeding of the minerals had been ongoing for seven months, affecting different growing phases of animals in Groups 1 and 2. Group 3 animals entered the farm after hazard identification (Fig. 1). Exposed animals had received only 60–70% of the calcium needed, but the amount of phosphorus was more than twice the recommended level. The Ca/P ratio was thus severely distorted.

In this study, we report post-mortem macroscopic findings of both front and hind limbs of the three groups of dairy bulls. At the time of slaughter, animals were clinically healthy. The first group of animals consisted of 20 bulls slaughtered between the ages of 18.3 and 19.4 (average 18.7) months. This group had received a low-calcium diet on average from the age of 9 months to 16 months. The second group consisted of 16 animals aged 21–22 (average 21.7) months. This group had received a low-calcium diet on average from the age of 4.6 months to 11.6 months. The third group consisted of 15 animal aged 18.3–20.9 (average 19.2) months; this group had a normal, mineral-balanced diet (Fig. 1).

#### Diet

During the feedlot period (200–600 kg bodyweight), fattening bulls are fed twice daily. Animals are divided into two feeding groups based on their estimated weight: those weighing 200–400 kg and those weighing over 400 kg. Feeding of the animals is based on a feeding plan (Table 1). The home-grown components of the diet (silage and barley) are analysed once a year. The feeding plan is also reviewed annually. The animals' diets consisted of barley (fresh preserved), ground rapeseed, mash and pre-dried hay silage. To satisfy mineral requirements, drinking water was supplemented with a balanced commercial preparation (Hiveblend^®^, Hiven Oy, Paimio, Finland). Calcium was mistakenly not given in the ration, and animals were therefore calcium-deficient for 8 and 7 months in Groups 1 and 2, respectively (Table 2).

**Table 1:** Target composition of the feeding plan diet.

Animal weight	200–400 kg	> 400 kg
Total amount of ME (MJ)	85.4	113.5
ME (MJ/kg DM)	11.7	11.7
% of roughage	40–60	40–60
% of crude protein	15.0	14.0
OIV (g/kg DM)	590	690
Calcium (g/d)	43	51
Phosphorus (g/d)	23	26
Ca/P ratio	1.8	1.8

ME = metabolizable energy

DM = dry matter

OIV = protein absorbable in small intestine

**Table 2:** Actual composition of diets. The calcium-deficient period is indicated with bold letters.

	Group 1			Group 2				Group 3		
Duration of diet (mo)	**4**	**4**	3	**6**	**1**	8	1	6	2	7
kg DM	**8.54**	**10.65**	11.22	**8.54**	**10.65**	11.22	10.15	7.48	11.22	10.15
Total amount of ME (MJ)	**96.53**	**120.39**	125.89	**96.53**	**120.39**	125.89	107.29	83.42	125.89	107.29
ME (MJ/kg DM)	**11.35**	**11.35**	11.23	**11.35**	**11.35**	11.23	10.53	11.12	11.23	10.53
% of roughage	**48.48**	**50.52**	49.56	**48.48**	**50.52**	49.56	40.43	53.05	49.56	40.43
% of crude protein	**13.66**	**13.19**	11.87	**13.66**	**13.19**	11.87	15.64	11.89	11.87	15.64
OIV (g/kg DM)	**91.59**	**86.76**	71.25	**91.59**	**86.76**	71.25	96.48	71.02	71.25	96.48
Calcium (g/d)	**29.89**	**34.81**	52.54	**29.89**	**34.81**	52.54	57.43	47.68	52.54	57.43
Phosphorus (g/d)	**48.05**	**54.97**	41.85	**48.05**	**54.97**	41.85	40.77	34.51	41.85	40.77
Ca/P ratio	**0.62**	**0.63**	1.26	**0.62**	**0.63**	1.26	1.41	1.38	1.26	1.41

ME = metabolizable energy

DM = dry matter

OIV = protein absorbable in small intestine

#### Sample collection and analyses

**Figure 2:**
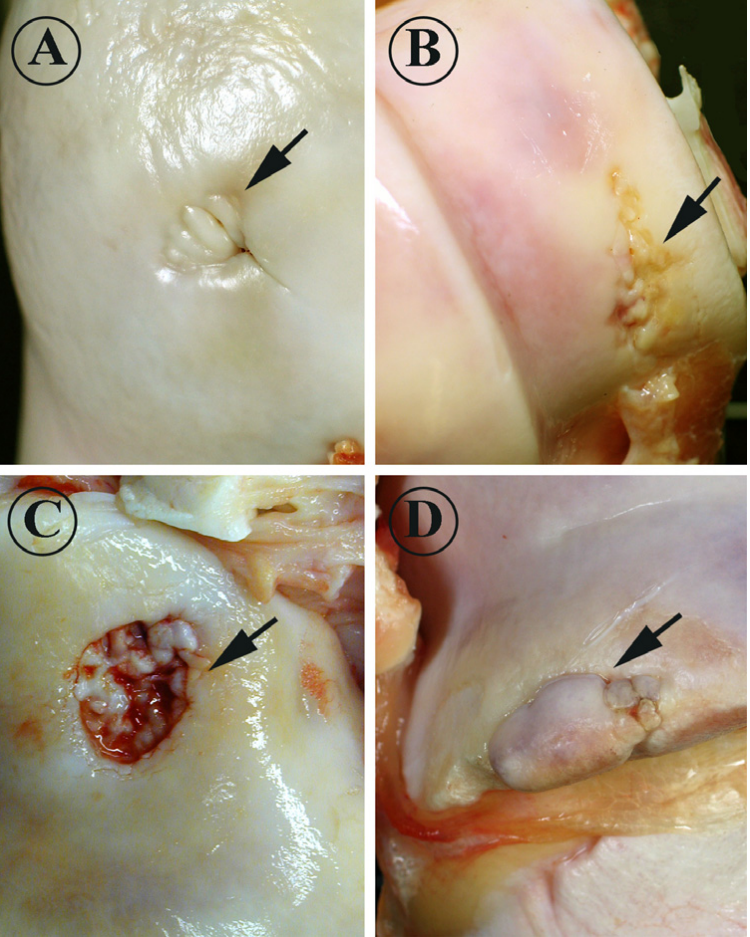
A: grade 1 osteoarthritic lesions had minor roughening of the articular-epiphyseal cartilage complex (AECC) and did not penetrate deeper than 2 mm (caput, femur). B: grade 2 lesions were moderate and penetrated 2-3 mm into the AECC (lateral trochlea, humerus). C: grade 3 lesions were severe and penetrated over 3 mm into the AECC (lateral condyle, tibia). D: grade 4 lesions were osteochondrosis dissecans-like lesions (lateral trochlea, femur).

The bones (scapula, humerus, radius, ulna, femur, tibia, fibula, talus, calcaneus) were removed at the abattoir. In Group 1, the bones were collected on a group basis. In Groups 2 and 3, the bones were collected on an animal basis. In Group 2, all scapulas were missed because of a sampling error. Bones were sent to the Section of Veterinary Pathology at the University of Helsinki, where they were stored below 4°C for 2–7 days prior to examination. All joint surfaces were evaluated macroscopically. Location, number and appearance of pathological changes were recorded. Changes were categorized into four grades (Fig. 2). Grade 1 was used when the lesion was minor, the joint surface was roughened, the articular cartilage was irregular and the lesion penetrated less than 2 mm into the articular cartilage (Fig. 2A). Grade 2 was used when the lesion was moderate, the joint surface was roughened and the changed area penetrated 2–3 mm into the articular cartilage (Fig. 2B). Grade 3 was used when the lesion was ulcerative and the change penetrated over 3 mm into the articular cartilage (Fig. 2C). Grade 4 was used for lesions classified as osteochondrosis dissecans (OD, Fig. 2D). The affected area was measured by using a round hole table (Faber-Castell 906c, Germany), with holes ranging from 1 mm to 36 mm. The depth of the affected area was also recorded.

#### Statistical methods

Differences in weight gain and income between the three groups were tested by one-way analysis of variance (ANOVA) followed by Tukey's test. Levene's test was used to evaluate the variance within each group. Chi-square test was used to explore differences between groups in the prevalence of severe lesions (lesions graded 2, 3 or 4). McNemar's test was applied to assess the difference between left and right leg bones. The effect of severe lesions in different locations on weight gain in Groups 2 and 3 was determined by t-test. Results are expressed as means or percentages (± standard errors of the mean (SE)). P-values of less than 0.05 were considered statistically significant.

## Results

The scapula was the bone most often affected (75%, Table 3). All OA lesions in scapulas were located in the glenoidal cavity, on its weight-bearing surface; 83% of the lesions were classified as grade 1 (Table 3).

**Table 3:** Number of affected bones and the location and grade of lesions.

			Number of lesions			
Bone	Site	% (a/n)	Grade 1	Grade 2	Grade 3	Grade 4
Scapula^x^	cavitas glenoidalis	75 (52/69)	45	7	0	0
Humerus	caput humeri	27 (27/99)	25	2	0	0
	medial trochlea	40 (40/99)	33	6	0	1
	lateral trochlea	8 (8/99)	3	2	0	3
Radius	fovea capitis radii	41 (40/97)	23	16	1	0
	incisura trochlearis	36 (35/97)	30	4	1	0
Ulna	processus anconeus	2 (2/97)	0	0	0	2
	processus coronoideus	2 (2/97)	0	0	0	2
Femur	caput femur	9 (9/98)	6	1	0	2
	trochlea ossis femoris	60 (59/98)	38	13	8	0
	medial condyle of trochlea	4 (4/98)	2	0	1	1
	lateral condyle of trochlea	4 (4/98)	0	1	0	3
Tibia	eminentia intercondylaris	1 (1/101)	0	1	0	0
	medial condyle	3 (3/101)	3	0	0	0
	lateral condyle	7 (7/101)	4	1	2	0
Tarsus	talus	73 (74/101)	37	23	2	12
	calcaneus	41 (41/101)	30	4	1	6

a = number of affected bones, n = number of examined bones

^x ^not available for Group 2

Predilection sites of OA lesions in the humerus were the medial trochlea (40% of affected bones) and the head of the humerus (27%). OA lesions in the medial trochlea could be divided into two locations; 66% of the changes were found in the medial ridge and 34% in the mid-region. However, the most severe lesions were situated in the lateral trochlea of the humerus.

Predilection sites of OA lesions in the head of the radius were the fovea capitis radii (42% of affected bones) and the incisura trochlearis (35%).

The predilection site of OA lesions in the femur was the trochlea ossis femoris (60% of affected bones). Lesions in the trochlea of femoris varied from a narrow, fissure-like vertical slit to a ≥ 10-mm crater-like ulceration and OD-like lesions (Fig. 2D). Lesions appeared to originate in the distal extremities of the trochlea, emerging vertically in the proximal direction.

The predilection site of OA lesions in the tibia was the lateral condyle.

The tarsal bones, specifically the talus and the calcaneus, were often affected; predilection sites were the articular surfaces between the tibia and these bones. Lesions in the talus were severe; 16% were classified as grade 4 (OD). The predilection site of OD lesions was the lateral trochlea of the talus.

OA lesions were commonly bilateral. Only 13.8% of lesions in the radius and 6.5% of lesions in the tarsus were unilateral. In the femur, 28.6% of lesions were unilateral (Table 4).

**Table 4:** Bilateralism of osteoarthritic lesions in Groups 2 and 3.

Bone	Bilateral lesion	Unilateral lesion	Bilaterally unaffected
Humerus	6.7	10.0	83.3
Radius	24.1	13.8	62.1
Femur	10.7	28.6	60.7
Tibia	0.0	3.2	96.8
Tarsus	41.9	6.5	51.6
Combined	51.6	16.1	32.3

**Figure 3:**
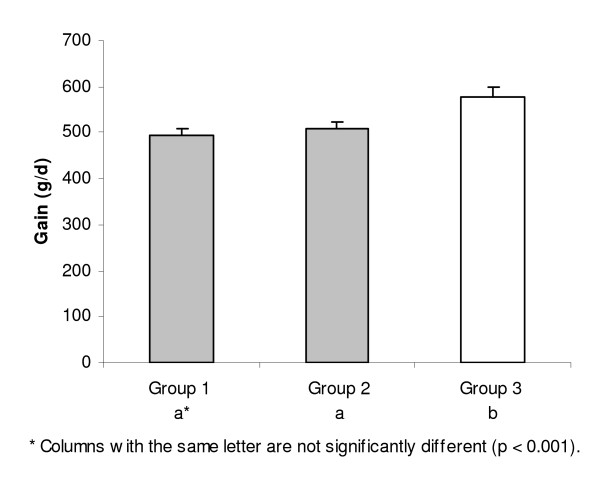
Comparison of daily weight gain (mean + SE). Calcium-deficient groups are indicated with shading.

The weight gain per day varied between groups (Fig. 3). Groups 1 and 2 had similar weight gains, but Group 3 had a significantly (P < 0.001) higher gain. Due to carcass classification on the EUROP.e system, animals in Group 3 produced 20% better income (mean 1.42 €/d, SE 0.06) than those in Group 2 (mean 1.19€/d, SE 0.04, P < 0.05) and over 30% better income than those in Group 1 (mean 1.08€/d, SE 0.05, P < 0.001). Incomes in groups 1 and 2 were not statistically different.

A significant difference was present between groups in the prevalence of severe lesions (lesions graded 2, 3 or 4). Groups with a low-calcium diet (Groups 1 and 2) had a significantly higher prevalence of OA lesions in the talus and trochlea ossis femoris (Table 5). When Groups 1 and 2 are compared, the incidence and severity of OA are higher in Group 2. These animals were calcium-deficient from the age of 5–12 months.

**Table 5:** Morbidity rate of severe osteoarthritic (OA) lesions (grade ≥ 2) in different locations in Groups 1–3.

		OA lesion grade ≥ 2				
Bone	Site	Group 1	Group 2	Group 3	Total	P-value
Scapula	cavitas glenoidalis	17.5	..	0	10.1	0.017*
Humerus	caput humeri	2.6	3.1	0	2	0.648
	medial torchlea	2.6	3.1	0	2	0.648
	incisura trochlearis	10.5	3.1	0	5.1	0.125
	medial trochlea (combined)	13.2	6.3	0	7.1	0.112
	lateral trochlea	2.6	12.5	0	5.1	0.058
Radius	fovea capitis radii	0	45.2	10.3	17.5	< 0.001***
	incisura trochlearis	8.1	9.7	0	6.2	0.247
Ulna	processus anconeus	2.7	3.2	0	2.1	0.64
	processus coronoideus	0	6.5	0	2.1	0.114
Femur	caput femur	0	12.9	0	4.1	0.011*
	trochlea ossis femoris	20.5	45.2	0	22.4	< 0.001***
	medial condyle of trochlea	2.6	3.2	0	2	0.652
	lateral condyle of trochlea	5.1	0	3.6	3.1	0.457
Tibia	eminentia intercondylaris	2.6	0	0	1	0.448
	medial Condyle	0	0	0	0	..
	lateral Condyle	5.1	3.1	0	3	0.46
Tarsus	talus	28.2	71.9	6.7	35.6	< 0.001***
	calcaneus	10.3	12.5	6.7	9.9	0.741

By studying only the femurs of the bulls, 40% of affected animals (animal having one or more severe OA lesions in any location) were detected. By combining the findings of femurs, tarsi and radii, the sensitivity was 100% (Table 6).

**Table 6:** Concurrence of lesions in different bones.

**Individual bone**	n*	n	S (%)	SE
Humerus	61	37	18.9	6.4
Radius	60	36	52.8	8.3
Femur	59	37	41.7	8.2
Tibia	62	37	2.7	2.7
Tarsus	62	37	75.7	7.1
**Combination of bones**				
Femur and humerus	58	36	58.3	8.2
Femur and tarsus	59	36	86.1	5.8
Femur and radius	57	35	77.1	7.1
Femur, tarsus and radius	57	35	100	0
**Combination of articular surfaces**				
Trochlea os femur, talus ja fovea capitis radii	57	35	88.6	5.4

n = Number of individual bones/legs, n* = Number of affected animals (animals with one or more osteoarthritic lesions graded ≥ 2). S = Sensitivity of examination of individual bones/combination of bones/combination of articular surfaces. SE = standard error of mean.

## Discussion

The faulty, heavily distorted dietary Ca/P ratio yielded a severe outbreak of OA in fattening bulls. Over 80% of the animals with a calcium-deficient diet had at least one severe OA lesion. However, OA lesions were prevalent also in animals with balanced diets, 30% of these animals having lesions.

Active discussion about the lameness of dairy cows and steering bulls is taking place worldwide. Reports indicate that as many as 60% of dairy cows show lameness at least once a year [16]. Lameness is also the third most common reason for early culling of dairy cows [17]. Steering bulls that are lame are unable to perform. On a beef farm, a lame steering bull can be a disaster. All of the above are reasons for farmers to pay special attention to the health of their animals. However, lameness in fattening dairy and meat bulls is often diagnosed at a fairly late stage. In modern husbandry, animals are kept in bigger groups, complicating the observation of individual animals. Lameness is frequently undetected until the animal can no longer stand or walk. Consistent with a previous report [5], OA lesions in our study were highly bilateral. Bilateral lameness is more difficult to detect. Only the most severe cases of OA tend to result in clearly visible lameness. The animals in our study were considered sufficiently healthy to be transported to the slaughterhouse; neither the owner nor the slaughterhouse veterinarian observed obvious signs of lameness. Animals were able to walk and put weight on each leg. However, OA lesions were abundant; over 80% of the animals with a calcium-deficient diet had at least one severe OA lesion. Even in the group with a balanced diet (Group 3), 30% of animals had at least one severe OA lesion. The question then arises that should pathological changes be considered a problem if the animal is not clinically lame? We believe that even subclinical OA in feedlot animals should be deemed a welfare problem.

To enhance animal welfare, it would be beneficial if economic losses were connected to the issue. How much does OA affect productivity? In the present case, the economic losses were obvious. At least six animals were euthanized or sent to the slaughterhouse earlier than planned due to acute lameness. Because of differences in net weight gain per day and in carcass classification on the EUROP.e system, animals in Group 3 produced 20% better income than animals in Group 2 and over 30% more money than animals in Group 1.

No significant association could be shown between OA lesions and the growth rate of animals. In Group 2, all animals had at least one severe OA lesion; thus, no case-control comparisons could be performed. In addition, Group 3 lacked sufficient cases for a case-control comparison. However, the overall trend does not rule out the existence of such a connection.

Predilection sites in bovine OC literature include stifle, hock, shoulder and elbow joints [5,7,10,18]. In the stifle joint, OC lesions are typically found in both the medial and the lateral trochlear ridge of the distal femur, in the patellar groove and in the medial intercondylar eminence of the tibia. In the hock joint, lesions have been reported mainly in the medial and the lateral condyle of the trochlea tali distalis, the lateral malleolus of the distal tibia and the coranoid process of the calcaneus [7]. In the shoulder joint, lesion sites are the central and dorsocranial areas of the humeral head and in the glenoidal cavity. In the elbow joint, lesions are frequently present on the articular surface of the distal radius [8,5]. The predilection sites for OA evaluated macroscopically in this study are consistent with previous reports of OC, indicating that many of the lesions observed here may origin from OC lesions. OA lesions were mainly found in the cavitas glenoidalis of the scapula, the head and the medial condyle of the humerus, the incisura trochlearis and the fovea capitis of the radius, the trochlea of the femur, the os talus and the os calcaneus. For a farm-level study, the most practical and convenient bone to investigate prevalence of OA and OC in dairy bulls is the femur.

Our findings suggest that calcium deficiency and mineral imbalance are predisposing factors for OA (Table 6). The incidence and severity of OA lesions being higher in Group 2 indicates that calcium deficiency has a more serious outcome in the age group 5–12 months than in the age group 12–18 months. Besides resulting in serious welfare problems, the animal groups with a suboptimal Ca/P ratio produced 30% less money than animals with an optimal Ca/P ratio. While diagnosing OA is difficult on the farm, OA lesions are fairly easy to spot in slaughterhouse material. Lesions in the trochlea of the femur are particularly easy to identify and measure. To control the growing problem of OA in feedlot farming, cooperation between the slaughterhouse and the farmer is essential.
